# CD133^+^ ovarian cancer stem-like cells promote non-stem cancer cell metastasis via CCL5 induced epithelial-mesenchymal transition

**DOI:** 10.18632/oncotarget.3462

**Published:** 2015-02-28

**Authors:** Haixia Long, Tong Xiang, Wei Qi, Jiani Huang, Junying Chen, Luhang He, Zhiqing Liang, Bo Guo, Yongsheng Li, Rongkai Xie, Bo Zhu

**Affiliations:** ^1^ Institute of Cancer, Xinqiao Hospital, Third Military Medical University, Chongqing, China; ^2^ Department of Obstetrics and Gynecology, Southwest Hospital, Third Military Medical University, Chongqing, China; ^3^ Department of Obstetrics and Gynecology, Xinqiao Hospital, Third Military Medical University, Chongqing, China; ^4^ Biomedical Analysis Center, Third Military Medical University, Chongqing, China

**Keywords:** cancer stem like cells, non-cancer stem like cells, chemokine (C-C motif) ligand 5, epithelial-mesenchymal transition, NF-κB

## Abstract

Cancer stem cells (CSCs, also called cancer stem-like cells, CSLCs) can function as “seed cells” for tumor recurrence and metastasis. Here, we report that, in the presence of CD133^+^ ovarian CSLCs, CD133^−^ non-CSLCs can undergo an epithelial-mesenchymal transition (EMT)-like process and display enhanced metastatic capacity *in vitro* and *in vivo*. Highly elevated expression of chemokine (C-C motif) ligand 5 (CCL5) and its receptors chemokine (C-C motif) receptor (CCR) 1/3/5 are observed in clinical and murine metastatic tumor tissues from epithelial ovarian carcinomas. Mechanistically, paracrine CCL5 from ovarian CSLCs activates the NF-κB signaling pathway in ovarian non-CSLCs *via* binding CCR1/3/5, thereby inducing EMT and tumor invasion. Taken together, our results redefine the metastatic potential of non-stem cancer cells and provide evidence that targeting the CCL5:CCR1/3/5-NF-κB pathway could be an effective strategy to prevent ovarian cancer metastasis.

## INTRODUCTION

Ovarian cancer is the most lethal gynecological cancer and ranks as the fifth most common cause of cancer-related death worldwide [1]. Approximately 70% of patients are diagnosed with either stage III or IV ovarian cancer accompanied by extensive intraperitoneal spread [2]. Understanding the mechanisms underlying the spread of ovarian carcinoma is critical to develop effective treatment strategies.

Previous studies have suggested that cancer stem cells (CSCs, also called initiating cancer cells or cancer stem-like cells), a small subset of cancer cells that can generate tumors through their self-renewal and multi-differentiation capacities, are essential for metastatic colonization [3]. Ovarian cancer, like many cancers, is driven by CSCs [4-7]. These cells are defined based on either differential expression of cell surface markers, such as CD133 [8], CD44 [9], CD117 [9] and CD24 [10], or differential biochemical properties, such as ALDH1 [7], and Side population [11-12]. We and other groups have reported that CD133^+^ ovarian cancer cells both from cell lines and primary tumors showed CSC characteristics, including self-renewal, expression of stem cell markers, multi-differentiation and tumor initiation capacities, and therefore defined these CD133^+^ cells as ovarian cancer stem-like cells (CSLCs) [8, 13-14].

Our group previously demonstrated that CD133^+^ ovarian CSLCs exhibit superior metastatic capacity compared with CD133^−^ non-CSLCs (NCSLCs) both *in vitro* and in a xenograft model [14]. Also, evidence from other studies supports the hypothesis that ovarian CSCs contribute more than non-CSCs to tumor metastases [1, 15]. These comparisons were made when CSCs and non-CSCs were separated into different fractions for functional examination. In contrast, under real pathological situations, metastatic tumors are heterogeneous; composed of multiple cells types, including both CSCs and non-CSCs. In the tumor microenvironment,under the influence of enriched inflammatory cytokines and chemokines, non-CSCs might have different properties than the cells cultured *in vitro*. Therefore, the metastatic characteristics of non-stem cancer cells should be redefined.

Epithelial cancer cells lose cellular polarity and cell-cell adhesions and gain metastatic and invasive properties by undergoing epithelial-mesenchymal transition (EMT) [16-20]. Cytokines and chemokines produced by tumor cells [16-17], cancer associated fibroblasts (CAFs) or tumor-associated immune cells can stimulate the EMT process and thereby promote cancer cell metastasis [18-20]. Our previous study demonstrated that a variety of metastasis related chemokines (including CCL5, SDF-1, CCL2 and CCL7) and cytokines (such as IL-1, IL-8, IL-23, S100B and BMP1) can be released from CD133^+^ ovarian CSLCs [14]. Some of them, such as IL-1 [21], IL-8 [22] and CCL2 [23], were previously shown to be involved in the EMT of cancer cells. Hence, we speculated that soluble mediators secreted by CD133^+^ ovarian CSLCs may not only maintain these cells' unique characteristics through autocrine signaling, but also induce CD133^−^ ovarian NCSLCs to acquire metastatic potential through a paracrine mechanism. The purpose of this study is to investigate the role of ovarian CSLCs in programming the mesenchymal transition of epithelial NCSLCs and the underlying signaling mechanism.

## RESULTS

### CSLCs enhance the metastatic capability of NCSLCs through the secretion of soluble mediators

**Figure 1:**
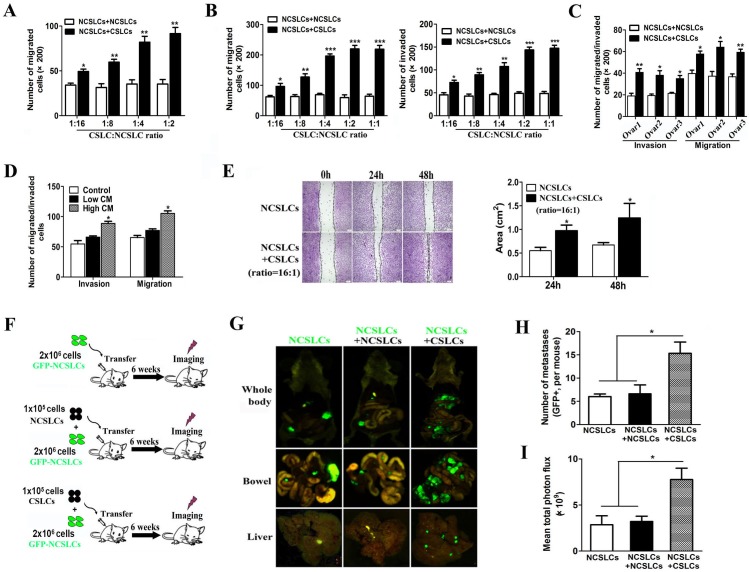
CSLCs promote NCSLC metastasis (A) 2×10^4^ RFP-labeled NCSLCs were seeded on the upper well (above the culture inserts) and cultured with either unlabeled NCSLCs or GFP labeled CSLCs at a 1:2, 1:4, 1:8, or 1:16 CSLC:NCSLC ratio, and the number of RFP+ NCSLCs that migrated was quantified. (B) Similar to (A), 2×10^4^ NCSLCs were plated on the upper well and indirectly co-cultured with NCSLCs or CSLCs in the lower chambers and the number of NCSLCs that migrated (left) or invaded (right) was quantified. (C) Similar to (B), NCSLCs were indirectly co-cultured with NCSLCs or CSLCs obtained from specimens from three different ovarian cancer patients and the number of NCSLCs that migrated or invaded was quantified. (D) NCSLCs were plated in the upper well in the presence of CM from high-density CSLCs (supernatant from 5×10^5^ CSLCs in 1 ml of medium), CM from low-density CSLCs (supernatant from 2.5×10^4^ CSLCs in 1 ml of medium), or control medium (medium incubated overnight without CSLCs) and the number of cells that migrated or invaded was quantified. (E) Wound healing assay for NCSLCs and NCSLCs co-cultured with CSLCs (ratio = 1:16). The migration into the gap at each time point was calculated and is shown in the graphs in the right panels. Scale bar = 50 μm. (F) Experimental model for investigating the effect of CSLCs on the metastasis of NCSLCs in vivo. (G-I) Biofluorescent images of the body (G top) and organs (G, bottom) of tumor-bearing mice. Scale bar = 75μm. The numbers of metastases per mouse are also shown (H). The fluorescent signal intensity was calculated and is shown in the graphs (I). The error bars represent the means ± standard deviation (SD) (n=6). Each experiment was repeated at least three times. *p<0.05, ** p<0.01, *** p<0.001.

To examine CSLC-NCSLC cross-talk in a mixed population of ovarian cells, in particular the potential role of CSLCs in promoting the migration and invasion capabilities of NCSLCs, we conducted a co-culture experiment. Employing our previously established protocol [14], CD133^+^ CSLCs and CD133^−^ NCSLCs were isolated from the A2780 ovarian cancer cell line and labeled with different fluorescence proteins by lentiviral transduction. NCSLCs expressing RFP were co-cultured with unlabeled NCSLCs (as control) or GFP labeled CSLCs at different ratios (Suppl. Fig. 1A), and the number of migrated RFP^+^ NCSLCs was quantified after co-culture for 24h. As shown in Fig. 1A and Suppl. Fig. 1B, the presence of CSLCs (at 1:16 to 1:2 ratios of CSLCs: NCSLCs) markedly promoted the migration and invasion of the RFP^+^ NCSLCs compared with control (co-cultured with unlabeled NCSLCs) (p < 0.05). To determine whether this effect was cell-cell contact dependent, we used a trans-well assay. We plated NCSLCs in the upper well and CSLCs or NCSLCs in the lower chambers (Suppl. Fig. 1A). We observed enhanced migration and invasion of NCSLCs when CSLCs were plated in the lower chambers, compared to when NCSLCs were plated in the lower chambers (p < 0.05, Fig. 1B and Suppl. Fig. 1C). Similarly, the migration and invasion of NCSLCs derived from primary ovarian cancer tissues was enhanced when CSLCs were plated in the lower chambers (p < 0.05, Fig. 1C and Suppl. Fig. 1D). These results indicated that CSLCs enhanced NCSLC migration and invasion, and this effect was dependent on CSLC-secreted mediators rather than on direct cell-cell contact.

To further investigate this issue, we cultured NCSLCs in conditioned medium (mixed at a 1:1 ratio with fresh medium) obtained from CSLCs or control medium with the same concentration of FBS (Suppl. Fig. 1E). We found that conditioned medium (CM) from CSLCs plated at high-density (supernatant from 5×10^5^ CSLCs in 1ml of medium) significantly promoted the migration and invasion of NCSLCs (p<0.05), whereas conditioned medium from CSLCs cultured at low-density (supernatant from 2.5×10^4^ CSLCs in 1ml of medium) did not (Fig. 1D and Suppl. Fig. 1F). Similarly, a wound healing assay showed that the presence of CSLCs in the upper well accelerated the migration of NCSLCs into the denuded area at 24h and 48h when compared with NCSLCs alone (p<0.05, Fig. 1E).

To determine the relevance of this *in vivo,* we utilized an established xenograft metastasis model (24) in which GFP-labeled NCSLCs alone, or mixed with unlabeled NCSLCs or CSLCs at a ratio of 1:20 (CSLCs:NCSLCs), were injected intraperitoneally (*i.p.*) into immunocompromised mice (Fig. 1F). Organs of the mice were dissected six weeks after implantation and fixed for pathological analysis. As shown in Fig. 1G-I, the bowels and livers of mice that received NCSLCs mixed with CSLCs displayed greater numbers of GFP^+^ metastases per mouse compared with those from mice bearing NCSLCs alone or mixed with unlabeled NCSLCs (p<0.05). Collectively, these results suggest that CSLCs enhance the invasion and metastasis of NCSLCs by secreting soluble mediators.

### CSLC-produced CCL5 enhances the metastatic capacity of NCSLCs through its receptors CCR1/3/5

**Figure 2:**
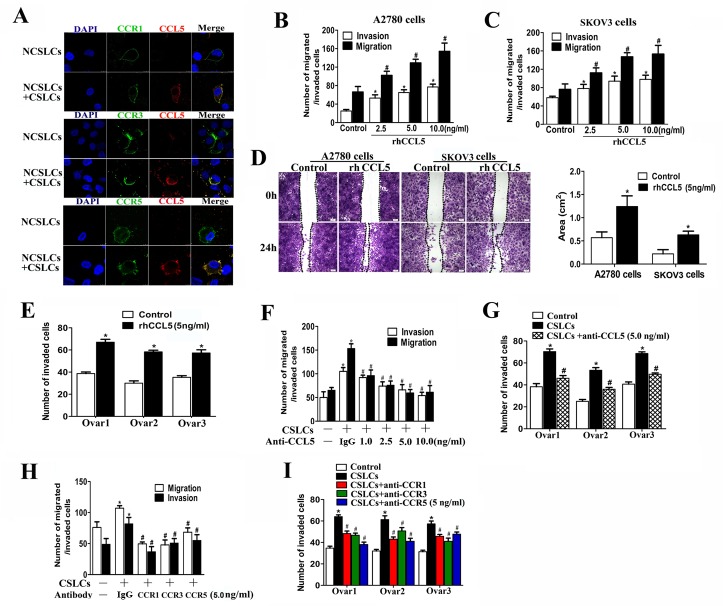
CSLCs enhance NCSLC metastasis through CCL5 (A) Confocal microcopy of NCSLCs and NCSLCs co-cultured with CSLCs (generated from A2780 cells) for 24 h and then stained with FITC-labeled anti-CCR1, anti-CCR3, and anti-CCR5 antibodies and CY3-labeled anti-CCL5 antibody. The cell nuclei were counterstained with DAPI. Scale bar = 25μm. (B and C) Transwell migration assay and matrigel invasion assay for NCSLCs derived from A2780 cells (B) and SKOV3 cells (C) in the presence of increasing concentrations of rhCCL5 (2.5-10 ng/ml). *p<0.05 for migration, #p<0.05 for invasion. (D) Wound-healing assay for NCSLCs derive from A2780 cells or SKOV3 cells in the presence or absence of rhCCL5 (5 ng/ml). The migration into the gap after 24 h was calculated and is shown in the graphs in the right panels. Scale bar =50μm. *p<0.05. (E) Similar to (B&C), NCSLCs generated from three ovarian cancer patients were treated with rhCCL5 (5 ng/ml), or left untreated, and the number of cells that invaded was quantified. *p<0.05. (F) Transwell migration assay and matrigel invasion assay for NCSLCs with or without CSLCs (generated from A2780 cells) plated in the lower wells in the presence or absence of anti-CCL5 antibody at 1-10 ng/ml. (G) Similar to (F), invasion assay for NCSLCs co-cultured with CSLCs (generated from specimens from three different ovarian cancer patients) in the presence or absence of anti-CCL5 antibody. (H) Similar to (F), NCSLCs co-cultured with CSLCs (generated from A2780 cells) in the presence or absence of anti-CCR1, anti-CCR3, or anti-CCR5 antibody (5ng/ml). (I) Similar to (H), NCSLCs co-cultured with CSLCs (generated from specimens from three different ovarian cancer patients in the presence or absence of anti-CCR1, anti-CCR3, and anti-CCR5 antibodies (5ng/ml). *p< 0.05 for the comparison between NCSLCs alone and NCSLCs co-cultured with CSLCs. #p< 0.05 for the comparison between NCSLCs co-cultured with CSLCs and NCSLCs co-cultured with CSLCs in the presence of indicated antibody. The error bars represent the means ± standard deviation (SD). Each experiment was repeated at least three times.

CCL5 produced by MSCs and CAFs in the tumor stroma has been reported to promote cancer metastasis [24-27]. Our previous study showed that this chemokine is abundantly produced by CSLCs and autocrine CCL5 signaling is indispensable for the invasive capability of CSLCs [14]. We were not able to detect CCL5 production from NCSLCs within the ovarian tumor mass or *in vitro* cell culture [14]. However, the receptors for CCL5, *i.e.* CCR1, CCR3, and CCR5, were expressed on the surface of NCSLCs to varying degrees [14, 26]. To confirm these results, we co-cultured A2780-derived NCSLCs with CSLCs for 24h. Consistent with previous reports [14, 26], some, but not all, CD133^−^ NCSLCs expressed CCR1, CCR3 or CCR5 (Fig. 2A). When CSLCs were present in the culture system separated by chambers, we observed CCL5 co-localization with CCR1, CCR3, and CCR5 on the membranes of NCSLCs (Fig. 2A).

To determine whether CCL5 affects the metastatic capacity of NCSLCs, we treated NCSLCs derived from either A2780 or SKOV3 with recombinant human CCL5 (rhCCL5). As shown in Fig. 2B and 2C, the treatment of NCSLCs with rhCCL5 (2.5-10 ng/ml) for 24 hours significantly enhanced their migration and invasion in a dose-dependent manner (p<0.05). Moreover, after treatment with 5 ng/ml of rhCCL5, both A2780 and SKOV3-derived NCSLCs migrated more rapidly to the denuded area in a wound healing assay compared with cells in culture media alone (Fig. 2D). In addition, 5.0 ng/ml rhCCL5 also significantly increased the invasion of NCSLCs derived from primary human ovarian cancers (Fig. 2E). Taken together, these results suggest that CCL5 significantly promotes the migration and invasion of NCSLCs, and suggest that CCL5 secretion may be involved in the enhancement of NCSLC metastatic potential by CSLCs.

To further validate this observation, we added an anti-CCL5 antibody to CSLC-NCSLC co-cultures derived from the A2780 cell line or from three primary human ovarian cancer tissues. Our results showed that neutralization of CCL5 reduced the number of invasive cancer cells in a dose-dependent manner (p<0.05, Fig. 2F and 2G). Similarly, blockade of the CCL5 receptors by antibodies against CCR1, CCR3, or CCR5 inhibited NCSLC migration and invasion (p<0.05, Fig. 2H and 2I). However, neither the CCL5 antibody nor antibodies to its receptors affected the invasion of NCSLCs in the absence of CSLCs (Suppl. Fig. 2).

**Figure 3:**
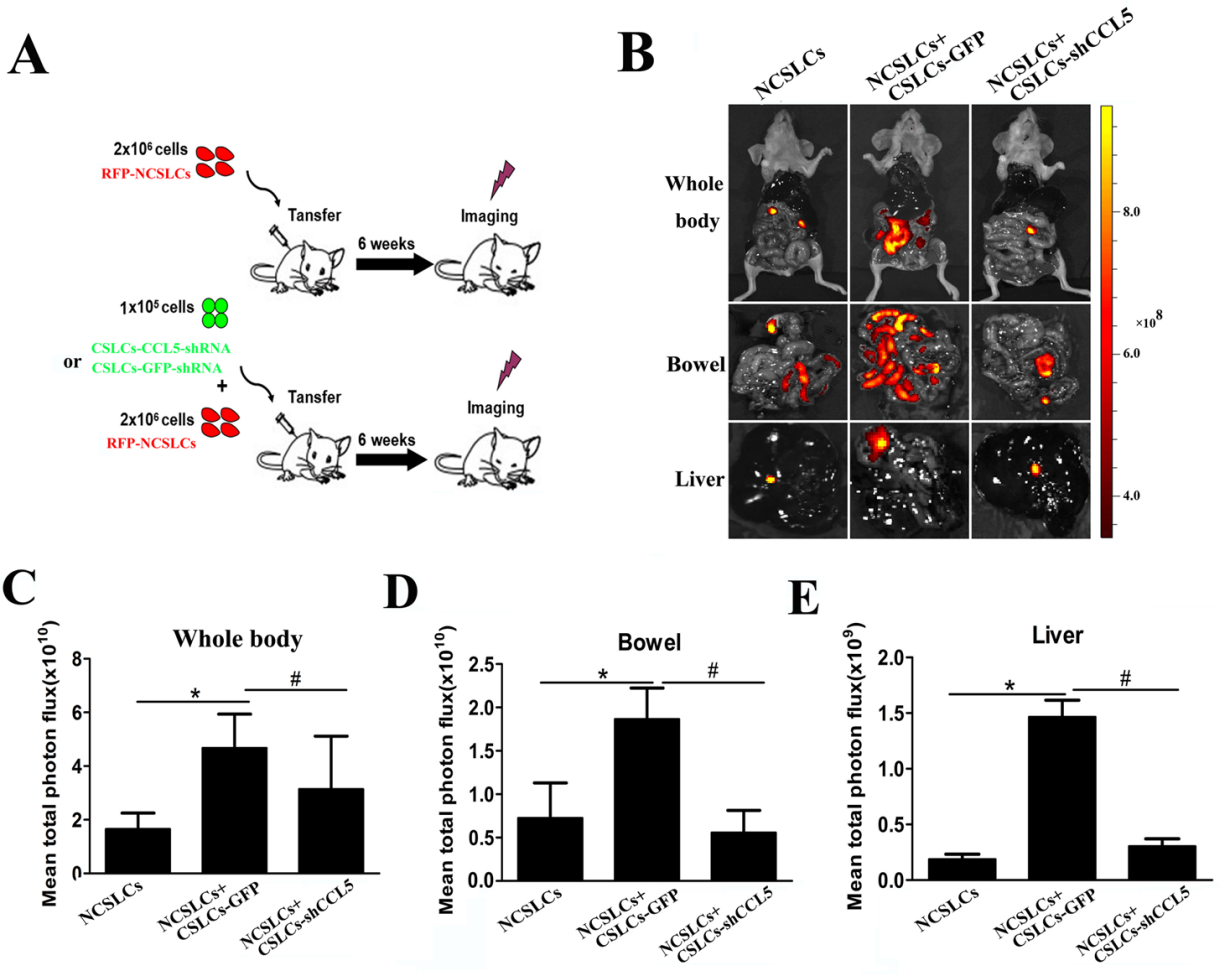
CSLC-produced CCL5 enhances NCSLC metastasis *in vivo* (A) Experimental model to investigate whether the promoting effect of CSLCs on the metastasis of NCSLCs is mediated by CCL5. RFP-labeled NCSLCs (2×10^6^) alone, or in combination with CSLCs (1×10^5^) transduced with lentivirus carrying GFP-shRNA or CCL5-shRNA, were injected intraperitoneally into SCID mice (6 mice/group). (B) Metastasis formation was examined by biofluorescence imaging. Fluorescent images of the whole body (top) and organs (bottom) of tumor-bearing mice are shown. (C-E) The fluorescent signal intensity for the indicated regions was calculated and is shown in the graphs. *p< 0.05 for the comparison between NCSLCs alone and NCSLCs in combination with CSLCs. #p< 0.05 for the comparison between NCSLCs with CSLCs transduced with lentivirus carrying GFP-shRNA and NCSLCs with CSLCs transduced with lentivirus carrying CCL5-shRNA. The error bars represent the means ± standard deviation (SD) (n=6).

To determine the effect of CCL5:CCR1/3/5 signaling on the enhancement of NCSLC metastasis by CSLCs *in vivo*, we utilized a short hairpin (sh) RNA which reduced the expression of CCL5 in CSLCs by ~75% (Suppl. Fig. 3). RFP-labeled A2780-derived NCSLCs alone, or NCSLCs mixed with CSLCs transduced with lentivirus carrying CCL5-shRNA, or lentivirus control, were injected *i.p.* into recipient mice, and tumor metastasis was then evaluated by *in vivo* imaging (Fig. 3A). The co-transfer of CSLCs expressing GFP significantly increased the number of NCSLC-derived metastatic nodules in recipient mouse organs, but this effect was diminished when CCL5 was knocked-down in CSLCs (Fig. 3B-E). Collectively, these data suggest that CSLC-derived CCL5 is critical for the enhancement of NCSLCs metastasis by CSLCs.

### Elevated expression of CCL5 and its receptors is associated with ovarian cancer metastasis

**Figure 4:**
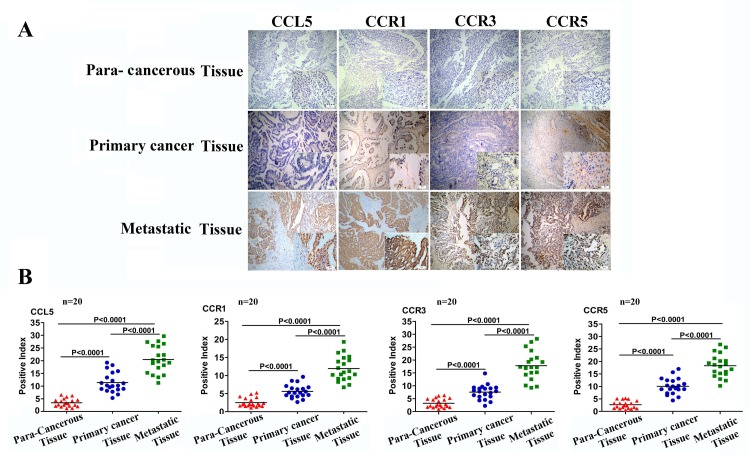
CCL5 and CCR1/CCR3/CCR5 expression is correlated with tumor invasiveness (A) Representative example of immunohistochemical staining for CCL5, CCR1, CCR3, and CCR5 in ovarian paracancerous tissue, ovarian primary cancer tissue, and ovarian metastasis tissue samples from the same ovarian cancer patient (n=20, paired patient specimens). Scale bar = 25μm. (B) The staining intensity in these sections was quantified according to histological scoring. The error bars represent the means ± standard deviation (SD) (n=20).

Previous studies have shown that myeloid-derived suppressor cells (MDSCs) [28], tumor-infiltrating lymphocytes (TIL) [29], MSCs [8], CAFs [26] and CSLCs [14] can produce CCL5 and that cancer cells express the CCL5 receptors CCR1, CCR3, and CCR5 [14, 26,30]. However, the clinical relevance of CCL5 to ovarian cancer progression, particularly metastasis, remains to be determined. To investigate this, we collected cancer tissues, corresponding metastatic tissues, and paracancerous tissues from 20 patients with epithelial ovarian cancer and determined the expression levels of CCL5 and its receptors (CCR1, CCR3, and CCR5). The results revealed that none of the CCL5, CCR1, CCR3 and CCR5 was expressed in the ovarian paracancerous tissues, whereas all these molecules were expressed in both primary cancer tissues and metastatic tissues (Fig. 4A). Compared with primary cancer tissues, the expression of both CCL5 and its receptors was two-fold higher in the metastatic cancer tissues (p<0.0001, Fig. 4B). These clinical analyses suggest that the CCL5 signaling is involved in ovarian cancer metastasis.

### EMT is involved in CSLC enhancement of NCSLC metastasis

**Figure 5:**
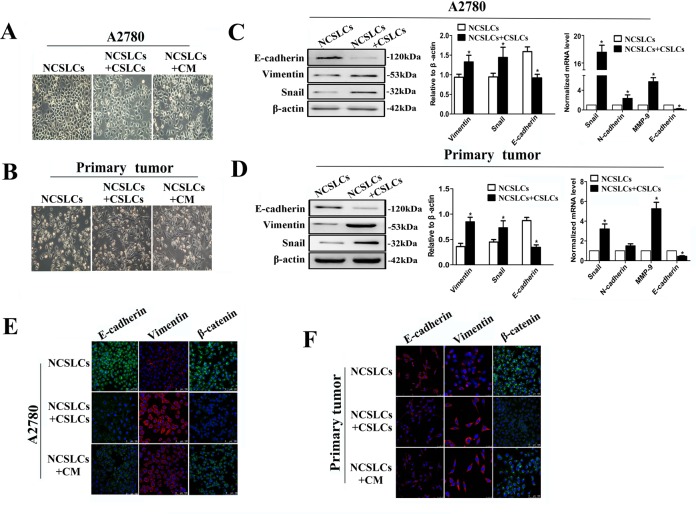
CSLCs induce EMT of NCSLCs (A and B) Phase-contrast images of NCSLCs cultured in the presence or absence of CSLCs, or conditioned medium (CM) from high-density CSLCs, for 48 h. The NCSLCs and CSLCs were generated from A2780 cells (A) or ovarian cancer patients (B). Magnification, 200×. C and D, EMT marker expression in NCSLCs cultured in the presence or absence of CSLCs. The markers were analyzed by Western blot (left) and qRT-PCR (right). β-actin was employed as a loading control for western blot and as a reference gene in qPCR. *p<0.05. The NCSLCs and CSLCs were generated from A2780 cells (C) or ovarian cancer patients (D). (E and F) EMT marker expression in NCSLCs treated with CM from high-density CSLCs, or co-cultured with CSLCs, or cultured with control medium. The markers were analyzed by immunofluorescence, and the nuclei were counterstained with DAPI. Scale bar=75 μm. The NCSLCs and CSLCs were generated from A2780 cells (E) or ovarian cancer patients (F). Scale bar=25μm. The error bars represent the means±standard deviation (SD). Each experiment was repeated at least three times.

To test whether the enhancement in the metastatic capability of NCSLCs induced by CSLCs was mediated by EMT, we examined the morphological characteristics of, and expression of EMT markers by A2780-derived NCSLCs after co-culture with A2780-derived CSLCs. NCSLCs cultured alone maintained their cobblestone-like morphology with strong cell-cell adhesions, while NCSLCs which were cultured with CM from high-density CSLCs or co-cultured with A2780-derived CSLCs at a ratio of 1:16 (CSLCs/NCSLCs) displayed a spindle-like morphology with fewer cell-cell contacts (Fig. 5A). A similar morphological transformation of NCSLCs in the presence of CSLCs was observed when both subsets were derived from primary ovarian cancer tissues (Fig. 5B). In addition, A2780- and primary human ovarian cancer-derived NCSLCs co-cultured with CSLCs consistently showed a significant reduction in expression of epithelial markers, such as E-cadherin and β-catenin; whereas the expression of mesenchymal markers, such as vimentin, snail, and MMP-9, were significantly increased, compared with NCSLCs cultured alone (p<0.05, Fig. 5C-F). Collectively, these results indicate that CSLCs can drive NCSLCs to undergo EMT.

### CCL5 mediates CSLC-induced EMT of NCSLCs

**Figure 6:**
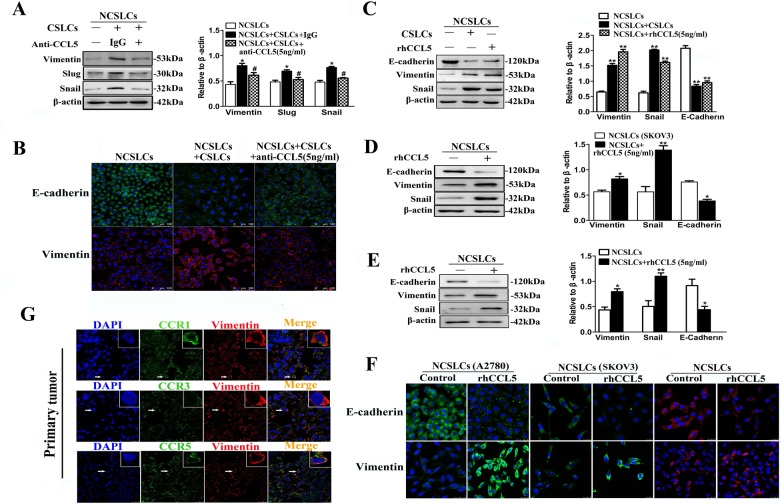
CCL5 mediates CSLC-induced EMT of NCSLCs (A and B) EMT markers in NCSLCs alone, or NCSLCs co-cultured with CSLCs in the presence or absence of anti-CCL5 antibody at 5ng/ml. The expression of EMT markers was analyzed by Western blot (A) and immunofluorescence (B). β-actin was employed as a loading control. *p< 0.05 for the comparisons between NCSLCs alone and NCSLCs co-cultured with CSLCs. #p< 0.05 for the comparisons between NCSLCs co-cultured with CSLCs in the presence and absence of the anti-CCL5 antibody. The NCSLCs and CSLCs were derived from A2780 cells, and the ratio of CSLCs:NCSLCs was 1:16. (C) Similar to (A), EMT markers on NCSLCs alone, NCSLCs co-cultured with CSLCs or NCSLCs cultured in the presence of 5 ng/ml rhCCL5. The expression of EMT markers was analyzed by Western blot. **p<0.01. The NCSLCs and CSLCs were derived from A2780 cells, and the ratio of CSLCs/NCSLCs was 1:16. (D and E) Similar to (C), EMT markers on NCSLCs generated from SKOV3 cells (D) or ovarian cancer patients (E) in the presence or absence of 5 ng/ml rhCCL5. The expression of EMT markers was analyzed by Western blot. *p<0.05, **p<0.01. (F) EMT markers on NCSLCs generated from A2780 cells, SKOV3 cells, or ovarian cancer patients in the presence or absence of 5 ng/ml rhCCL5. The expression of EMT markers was analyzed by immunofluorescence. Scale bar=25 μm. (G) Confocal microscopy of ovarian cancer tissue sections stained *in situ* with anti-CCR1/CCR3/CCR5 antibodies and anti-vimentin antibody. The cell nuclei were counterstained with DAPI. Scale bar=25μm. The error bars represent the means±standard deviation (SD). Each experiment was repeated at least three times.

We next sought to determine the effect of CCL5 on the EMT of NCSLCs. As shown in Fig. 6A, the addition of anti-CCL5 antibody to the A2780 derived NCSLC:CSLC co-culture system inhibited the CSLC-induced increase in expression of the mesenchymal markers vimentin, snail, and slug (p<0.05). Immunofluorescence confirmed that anti-CCL5 antibody inhibited CSLC-induced vimentin induction and E-cadherin down-regulation in this system (Fig. 6B). Conversely, the incubation of A2780-derived, SKOV3-derived, or primary ovarian cancer derived NCSLCs with rhCCL5 decreased the level of the epithelial marker E-cadherin and increased the level of the mesenchymal markers vimentin and snail (Fig. 6C-F). We next decided to investigate whether other cancer cell lines also showed similar responses to rhCCL5. Two lung adenocarcinoma cell lines (A549 and NCI-H1650) also showed a similar increase in mesenchymal markers and decrease in epithelial markers in response to rhCCL-5 (Suppl. Fig. 4-5). However, although a high proportion of MDA-MB-231 cells expressed CCR1/CCR3/CCR5 (Suppl. Fig. 4), rhCCL5 had no effect on the expression of the mesenchymal markers E-cadherin, vimentin and snail by MDA-MB-231 cells (Suppl. Fig. 5), consistent with a previous report [24]. Importantly, cancer cells in human primary tumor tissues that were CCR1, CCR3 or CCR5-positive expressed high levels of vimentin (Fig. 6G). These results indicate that CCR1/CCR3/CCR5-positive cancer cells undergo an EMT-like process in the presence of CSLCs.

### The NF-κB signaling pathway is involved in CCL5-enhancement of NCSLC migration

**Figure 7:**
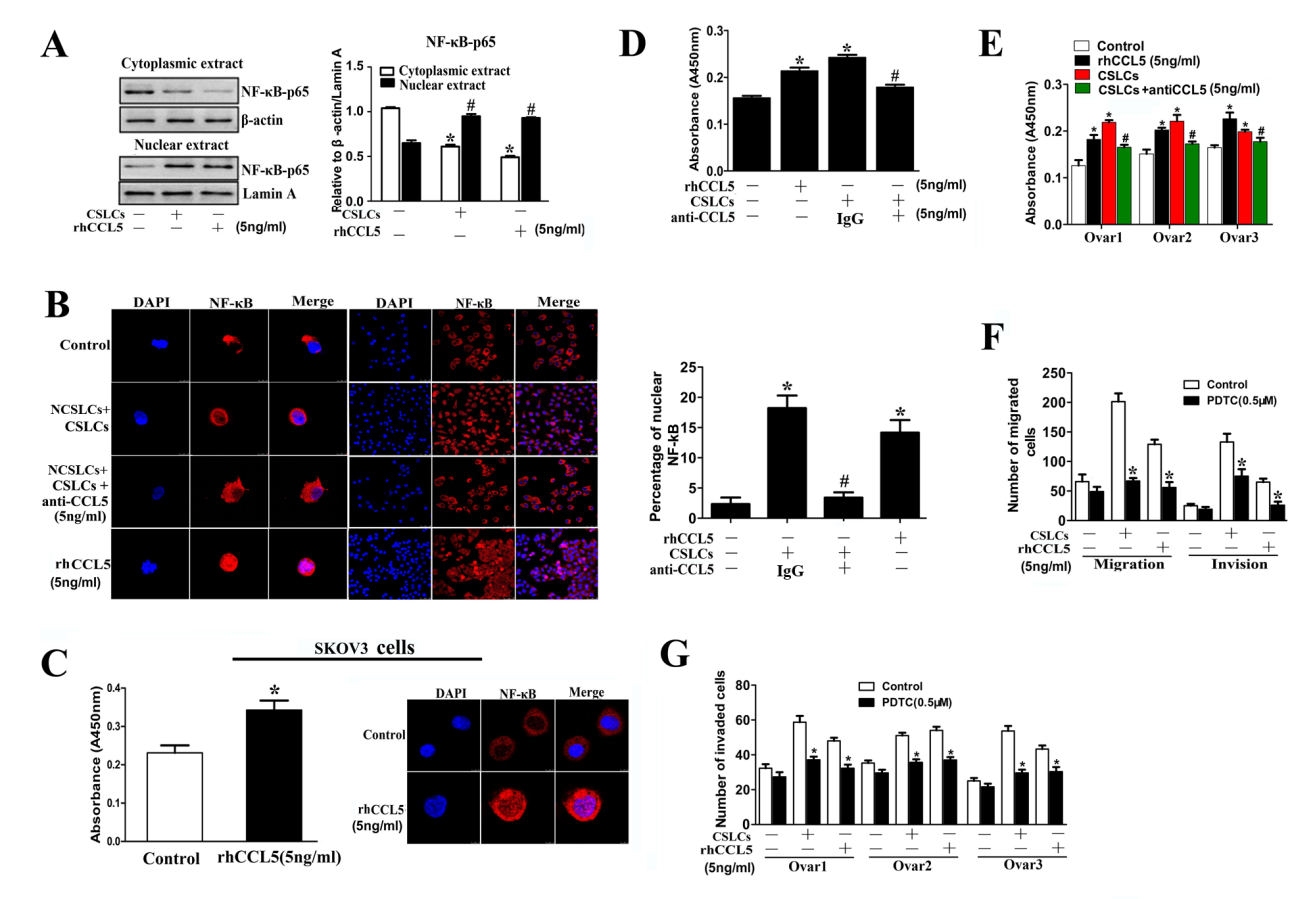
The NF-κB signaling pathway is involved in ovarian CSLC-mediated enhanced migration of NCSLCs (A) Western blot analysis of the expression of the NF-κB p65 subunit in NCSLCs, NCSLCs co-cultured with CSLCs or NCSLCs directly stimulated with rhCCL5 (5 ng/ml). β-actin was employed as the loading control for the cytoplasmic fraction, and Lamin A was used to normalize the loading of the nuclear fraction. *p<0.05 for the comparisons between the cytoplasmic fractions. #p<0.05 for the comparisons between the nuclear fractions. (B) NCSLCs derive from A2780, NCSLCs directly stimulated with rhCCL5, and NCSLCs co-cultured with CSLCs in the presence or absence of anti-CCL5 antibody were stained with phospho-p65 antibody and then with Cy3-conjugated secondary antibody (red). The cell nuclei were counterstained with DAPI (left). The percentage of cells with nuclear staining of phospho-p65 was calculated from the positively stained cells (right). *p < 0.05 for the comparisons between NCSLCs alone and NCSLCs co-cultured with CSLCs or NCSLCs directly stimulated with rhCCL5. #p<0.05 for the comparisons between NCSLCs co-cultured with CSLCs in the presence or absence of anti-CCL5 antibody (5ng/ml). (C) NF-κB activity and nuclear localization in NCSLCs (generated from SKOV3 cells) and NCSLCs directly stimulated with rhCCL5 was measured by ELISA and immunofluorescence. *p<0.05. (D and E) NF-κB activity in NCSLCs, NCSLCs co-cultured with CSLCs and NCSLCs directly stimulated by rhCCL5 was measured by ELISA (D, cells generated from A2780; E, cells generated from three ovarian cancer patients). *p<0.05 for the comparisons between NCSLCs alone and NCSLCs co-cultured with CSLCs or NCSLCs directly stimulated with rhCCL5 (5 ng/ml). #p<0.05 for the comparisons between NCSLCs co-cultured with CSLCs in the presence or absence of anti-CCL5 antibody (5ng/ml). (F) The migration and invasion capabilities of NCSLCs derived from A2780, NCSLCs co-cultured with CSLCs or directly stimulated with rhCCL5 in the presence or absence of PDTC (0.5 μM). *p<0.05 for the comparisons between with or without PDTC. (G) Similar to (F), the invasion capabilities of NCSLCs, NCSLCs co-cultured with CSLCs (generated from three ovarian cancer patients) or directly stimulated with rhCCL5 in the presence or absence of PDTC (0.5μM). *p<0.05 for the comparisons between with or without PDTC. The error bars represent the means±standard deviation (SD). Each experiment was repeated at least three times.

We previously found that NF-κB activation was necessary for the CCL5-mediated maintenance of the metastatic capability of CSLCs [14]. Moreover, a variety of studies have shown that NF-κB activation, through regulation of Twist expression and stabilization of Snail, promotes the EMT program in cancer cells [31-35]. Therefore, we speculated that the promotion of NCSLC EMT by CSLC-derived CCL5 may be mediated through the NF-κB signaling pathway. We found that NCSLCs co-cultured with CSLCs, or treated with rhCCL5, showed increased nuclear translocation of NF-κB-p65 compared with NCSLCs cultured alone (Fig. 7A-C). Moreover, the ELISA results showed that either co-culture with CSLCs or rhCCL5 treatment could induce NF-κB activation in NCSLCs (p<0.05, Fig. 7C-E). To address whether CCL5 induces NF-κB activation in NCSLCs, the anti-CCL5 antibody (at 5ng/ml) was added into the co-culture (NSCLCs+CSLCs) system. We found that neutralization of CCL5 significantly reduced NF-κB activity caused by CSLCs co-culture, in both A2780-derived (p<0.05, Fig. 7D) and human ovarian cancer tissue-derived NCSLCs (p<0.05, Fig. 7E). These results suggest that the CSLC-induced activation of NF-κB in NCSLCs is mediated by CCL5.

To determine the role of the NF-κB signaling pathway in the enhancement of the metastatic capacity of NCSLCs by CSLC-derived CCL5, we used pyrrolidine dithiocarbamate (PDTC, an inhibitor of NF-κB) to inhibit NF-κB activation in NCSLCs co-cultured with CSLCs or in NCSLCs treated with rhCCL5. As expected, the enhanced migration and invasion of NCSLCs induced by CSLC co-culture or rhCCL5 treatment was significantly reduced by the addition of an NF-κB inhibitor (p<0.05, Fig. 7F and 7G). These results suggest that the NF-κB signaling pathway is involved in enhancement of NCSLC metastasis by CSLC-derived CCL5.

## DISCUSSION

Since CSCs in epithelial ovarian cancer were discovered, and their superior migration and invasion capabilities, compared to NCSLCs, were identified, the contribution of NCSLCs to tumor recurrence and metastasis has been largely overlooked [1,14]. Moreover, the conversions between CSCs and non-stem cancer cells are of great interest. Recent studies provided conclusive evidence that, at least in the case of breast cancer, non-stem cancer cells can dedifferentiate to gain stem cell-like properties, both spontaneously *in vitro* and in a mixed xenograft with CSCs *in vivo* [36]. The plasticity of non-stem cancer cells makes it possible that these cells contribute to tumor metastasis. In this study, we reported a novel mechanism through which CD133^+^ ovarian CSLCs promote metastases derived from CD133^−^ NCSLCs by inducing EMT. Although we have not established whether these cells that have undergone an EMT-like process have a stem cell-like phenotype, our study provided a direct model to demonstrate the participation of non-stem cancer cells in ovarian cancer metastasis.

During the last decade, CD133^+^, CD44^high^ CD24^−^, CD44^+^CD117^+^ and ALDH^+^, together with side population, were identified as phenotypic and biochemical markers of ovarian CSCs, and have been used for identification of ovarian CSCs [8-12]. Ovarian CSCs isolated by whichever method have several characteristics of stem cells. Several studies have shown that CD133^+^ ovarian cancer cells are more tumorigenic and more resistant to chemotherapeutic treatment than CD133^−^ ovarian cancer cells within a heterogeneous tumor mass [8, 13-14, 37-38], even though tumor-initiating cell activity was also found in the CD133- fraction. We have previously reported that CD133^+^ ovarian CSLCs, which are endowed with a high metastatic capability, could produce substantial CCL5, whereas the CD133^−^ fraction has lower metastatic potential and did not produce detectable CCL5 [14]. In this study, we found that CD133^−^ ovarian cancer cells had enhanced metastatic potential in the presence of the CD133^+^ population. Therefore, our present study together with previous findings suggest that CD133^+^ ovarian cancer cells are distinct from the CD133^−^ population in terms of metastatic capability and CCL5 production, but the former can enhance the metastatic capacity of the latter.

CCL5 has been defined in several cancers as a pro-malignant factor that correlates with poor prognosis and elevated metastasis in tumor patients [39-40]. CCL5 produced by MSCs within the tumor stroma plays a crucial role in breast cancer cell invasion [24]. In addition, it has been reported recently that CCL5 is one of the most highly upregulated chemokines when CAFs are reprogrammed in epithelial ovarian cancer [26]. Our present results showed that CCL5 produced by ovarian CSLCs could promote cancer cell metastasis by inducing EMT. In ovarian cancer, MSCs comprise ~6% of cells in human ovarian tumor ascites, however, the percentage of MSCs in solid tumors is much lower (~0.3%) [41]. CAFs, comprising ~15% in ovarian cancer, are a major constituent of the tumor stroma [42]. CSCs comprise 1%-5% of cells in human ovarian tumor ascites or solid tumors [43]. Given the relative percentages of these cell types, one could argue that CAFs may play a more important role than CSCs or MSCs in CCL5-mediated ovarian cancer metastasis. However, the relative levels of CCL5 produced by CSCs, MSCs and CAFs remain to be determined. Moreover, in tumors after chemotherapy CSCs are enriched, with some cases displaying a ~9.5 fold increase in percentage such that CSCs constitute ~30% of cells in the tumor [44]. Therefore, the main source of CCL5 in the tumor, and the relative contribution of the various cell types to CCL5-mediated ovarian cancer metastasis are likely dependent on each individual patient's status.

EMT plays a critical role in tumor metastasis and recurrence, which is tightly linked with the biological specification of CSCs [45-46]. A direct molecular link between EMT and a stem cell-like phenotype was demonstrated by findings that EMT activators, such as Twist1 and ZEB1, can co-induce EMT and stem cell-like properties [47-48]. It has also been proposed that NCSCs may be converted into CSCs through EMT induction [48-50]. Therefore, we speculate that EMT of NCSLCs represents an intermediate step towards conversion into a stem cell-like phenotype. Nevertheless, this transition stage is critical and may be sufficient for secondary tumor formation by NCSLCs.

In the present study, the introduction of ovarian CSLCs significantly enhanced the EMT progression of NCSLCs. We also observed that the enhanced metastatic capacity of NCSLCs induced by CCL5 is associated with elevated EMT in both A2780 and SKOV3 ovarian tumor models and in two non-small cell lung cancer cell lines (A549 and H1650). However, we did not observe increased EMT of MDA-MB-231 cells in response to CCL5, consistent with the results from Karnoub and colleagues [24]. MDA-MB-231 breast cancer cells are reported to be more mesenchymal-like because these cells express high levels of mesenchymal markers, such as vimentin, ﬁbronectin, and slug, whereas the epithelial marker E-cadherin was barely detectable [50]. These results indicate that the EMT-promoting effect of CCL5 is much more prominent in epithelial-phenotype tumor cell lines, such as A2780, SKOV3, A549, and H1650.

We and others have reported that CD133^+^ CSLCs represent 0.1% to 3% of the total tumor mass in ovarian cancer [8, 13-14]. Since this is a minority population, it seems reasonable to speculate that the paracrine-dependent EMT induction in NCSLCs by CSLCs should be limited to cells within a short distance of the CSLCs. In contrast, this population can be greatly enriched in tumors after chemotherapy because CSCs are more drug-resistant [44-51]. Hence, our present findings suggests that chemotherapy and radiotherapy may not stop and might somehow promote cancer metastasis, since chemotherapy and radiotherapy could enrich CSCs [3, 52], which have a great capacity for metastasis and can induce the conversion of remaining chemo- or radio-resistant non-stem cancer cells into EMT-like cells. Therefore, there is an urgent need to develop a strategy that targets CSCs to prevent relapse and control metastasis.

To summarize, in this study, we demonstrated that ovarian CSLCs significantly promoted the migration and invasion of NCSLCs both *in vitro* and *in vivo*. This process is dependent on the CCL5:CCR1/CCR3/CCR5-NF-κB signaling axis (Suppl. Fig. 6). To our knowledge, this is the first evidence to demonstrate that CCL5 secreted by CSLCs is a potent initiator for EMT in NCSLCs. The discovery of a cross-talk between CSLCs and NCSLCs provides a molecular and cellular basis to explain why chemotherapy and radiotherapy can shrink primary tumors but cannot prevent distant metastases.

## MATERIALS AND METHODS

### Generation and culture of ovarian CSLCs and NCSLCs from cell lines and primary tumor tissues

Human ovarian cancer cell lines A2780 and SKOV3, lung cancer cell lines NCI-H1650 and A549, and the breast cancer cell line MDA-MB-231 were purchased from the American Type Culture Collection (ATCC). These cell lines were last tested by short tandem repeat profiling in September, 2012. The 22 fresh tumors used in this study were categorized as stage III serous ovarian adenocarcinomas. Primary ovarian cancer cells were isolated from freshtumor samples using cancer cell isolation kit (Panomics, CA) according to the manufacturer's instruction and previously published paper [53]. Then CD133^+^ CSLCs and CD133^−^ NCSLCs were isolated from purified cancer cells by FACS (AC133-PE, mouse IgG, Miltenyi) as described previously [14]. All studies on the enrolled patients were performed using protocols approved by the institutional review board of the Third Military Medical University and written informed consent was obtained from each patient. To establish stable CSLCs with reduced levels of CCL5, CSLCs were transduced with lentivirus carrying CCL5-shRNA (or GFP-shRNA as the control), as described previously [14].

### Collection of conditioned medium (CM)

CSLCs were seeded at a density of either 5×10^5^ (high) or 2.5×10^4^(low) cells/ml in Dulbecco's Modified Eagle Medium (DMEM) without FBS or other additives. 24 hours later, CM was collected, filtered through a 0.2 μm filter and stored at −80 °C. Medium collected from a simultaneous incubation without CSLCs served as a control. CM or control medium mixed with fresh medium at a 1:1 ratio was used in experiments as described [54].

### *In vivo* xenograft experiments

Female severe combined immunodeficient (SCID) mice were purchased from the Chinese Academy of Medical Sciences (Beijing, People's Republic of China). The mice were housed and maintained in laminar flow cabinets under specific pathogen-free conditions. For the xenograft experiments, GFP-labeled A2780-derived NCSLCs (2×10^6^) alone or in combination with CSLCs (1×10^5^), or RFP-labeled A2780-derived NCSLCs (2×10^6^) alone or in combination with CSLCs (1×10^5^) infected with lentivirus carrying GFP-shRNA or CCL5-shRNA were injected intraperitoneally into the recipient mice (n=6 mice/group). The body and organs of the mice bearing ovarian tumors were examined using biofluorescence imaging. The tumor xenograft and organs of the mice were harvested for further evaluation by qRT-PCR, Western blot, immunofluorescence, and H&E staining. The care and use of mice was performed in accordance with the local ethical guidelines. Each experiment was repeated at least three times.

### Immunohistochemistry and immunoﬂuorescence

Human ovarian tumor samples categorized as serous ovarian adenocarcinomas were obtained from 20 female patients (median age, 56.1 years; range, 26-69) at Xinqiao Hospital of the Third Military Medical University from 2009 to 2011. Formalin-fixed paraffin-embedded ovarian cancer tissues were cut into 3-μm-thick sections. The specimens were incubated with antibodies against CCL5, CCR1, CCR3, and CCR5 (BD Biosciences). All of the immunohistochemical photomicrographs were analyzed using Image Pro Plus (IPP, version 6.0). The positive index was determined by counting the percent of the positive cells in total cells using 400 × magnification, which was calculated in 5 random visions (microscopic fields?) by two pathologists and analyzed by Graphpad software, according to the methods described previously [1]. All of the protocols used in this study were approved by the institutional review board of the Third Military Medical University and written informed consent was obtained.

The immunofluorescence analysis was performed on 8-μm-thick frozen sections that were fixed with ice-cold 4% paraformaldehyde for 15 minutes at 37°C, blocked with normal serum for 20 minutes at room temperature, and incubated with one or more specific antibodies against vimentin, E-cadherin (1:200, Santa Cruz Biotechnology), CCR1, CCR3, CCR5 (1:100, BD Biosciences), or NF-κB (1:200, BD Biosciences) overnight in dark at 4°C. After three washes, the slides were stained with FITC- or Cy3-conjugated antibodies (Abcam). The nuclei were counterstained with DAPI. The stained cells were visualized with an Olympus confocal microscope. Each measurement was performed in triplicate.

### Western blotting

The preparation of cell lysates and the Western blot analyses were performed as described previously (14). The Nuclear-Cytosol Extraction Kit (KEYGEN) and Phospho-Protein Purification Kit (KEYGEN) were used for the extraction of nuclear and cytoplasmic protein. Antibodies against E-cadherin, snail, vimentin (1:500, Santa Cruz Biotechnology), NF-κb (p65) (1:200, BD Biosciences), and β-actin (1:400, Boster) were used. Each experiment was repeated at least three times.

### RNA extraction and quantitative real-time PCR analysis

qPCR was performed as described previously (14). The primers for human CCL5, CCR1, CCR3, CCR5, E-cadherin, snail, vimentin, matrix metalloproteinase-9 (MMP-9), and β-actin are listed in Suppl. Table 1. The qRT-PCR analysis was performed using an ABI7500 Prism Sequence Detection System (Applied Biosystems) with a SYBR Green kit (TAKARA). Each measurement was performed in triplicate. The relative gene expression levels were calculated using the comparative Ct (ΔΔCt) method, with β-actin as a reference gene.

### *In vitro* wound healing migration assay

NCSLCs were seeded on six-well culture plates (1×10^6^ cells/well) to near confluence, and the cells were treated with mitomycin C for 2 hours to inhibit cell proliferation prior to the assay. A linear wound was carefully produced by scraping a 20 μl sterile pipette tip across the confluent cell monolayer, and the cell debris was washed off with PBS. Cells were then treated with 5.0 ng/ml of rhCCL5, or co-cultured with CSLCs (1×10^5^ cells/well) in the upper well, in Dulbecco's modified Eagle's medium containing 1% fetal bovine serum. The wounded monolayers were stained with crystal violet and then photographed at 0, 24 and 48 h after wounding. Quantitative analysis of the wounds was performed by digitally capturing the wounded area using Scion Image (Scion Corporation, Frederick, MD) software as described [54]. Each experiment was repeated at least three times.

### Enzyme-linked immunosorbent array (ELISA)

The preparation of the supernatants was performed as described previously (14). ELISA for NF-κB activity was performed in accordance with the manufacturers' instructions (NF-κB/p65Active ELISA assay, Cayman). Each measurement was performed in triplicate.

### Statistical analyses

All of the data are presented as the means ± SD. The statistical analyses were performed using Chi-square test, Student's t test or one-way ANOVA. A difference was considered statistically significant when p <0.05. All of the statistical analyses were performed using the SPSS 13.0 software.

## SUPPLEMENTARY FIGURES


