# Supplementary material for: CD133+ ovarian cancer stem-like cells promote non-stem cancer cell metastasis via CCL5 induced epithelial-mesenchymal transition
Source: Oncotarget. 2015 Feb 28;6(8):5846–59. doi: 10.18632/oncotarget.3462 (PMC4467406; doi:10.18632/oncotarget.3462)
Supplement: Supplementary file 1 [file oncotarget-06-5846-s001.pdf]

CD133+ ovarian cancer stem-like cells promote non-stem cancer cell metastasis via CCL5 induced epithelial-mesenchymal transition

Supplementary Material

Supplementary figure 1

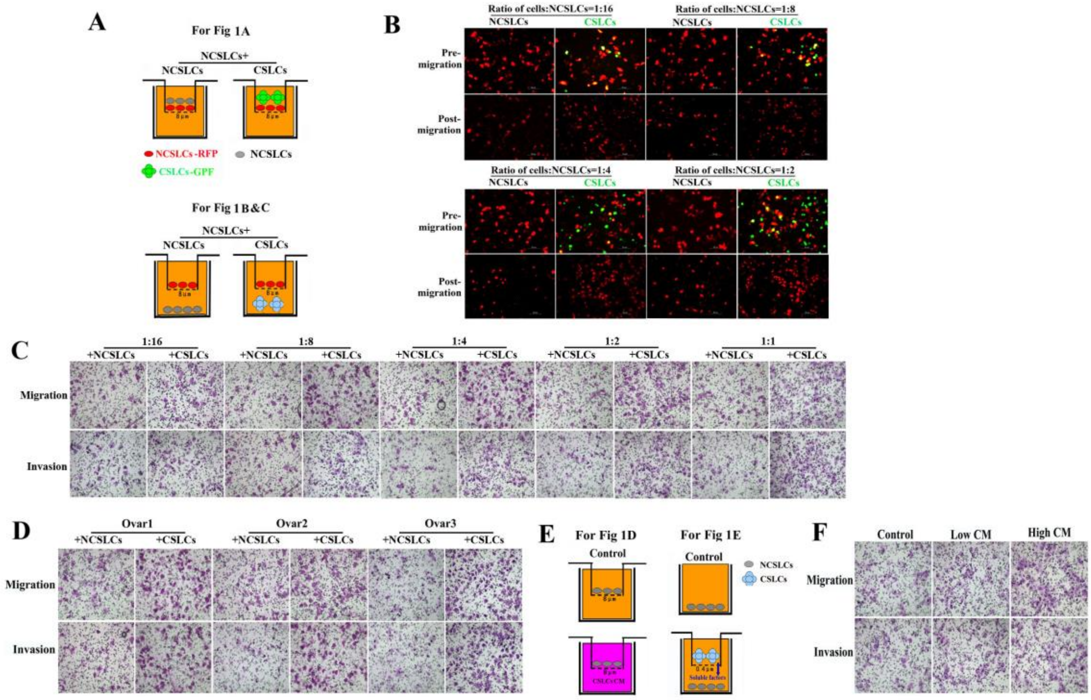

Supplementary Figure1: Migratory capacities of non-CSLCs cultured with CSLCs or the culture medium from CSLCs.

Related to figure 1. (A) Schematic representing the experimental protocols of direct and indirect co-culture of NCSLCs with CSLCs. (**B**) Migratory capacities of RFP-labeled NCSLCs cultured with CSLCs at a ratio of 1:2, 1:4, 1:8 or 1:16. Pre-migration cells (GFP labeled CSLCs or non-labeled NCSLCs and RFP-labeled NCSLCs, on top) and post-migration cells (only show RFP-labeled NCSLCs, on bottom). (**C and D**) Migratory and invasion capacities of NCSLCs indirectly co-cultured with CSLCs plated in the lower chambers. The cells were derived from A2780 cells (**C**) or ovarian cancer patients (**D**). **(E)** Schematic representing the experimental protocol for culture of NCSLCs with conditioned medium (CM) from CSLCs, or for wound healing assay. (**F**) Migratory and invasion capacities of NCSLCs, plated in the upper well, in the presence of CM from high-density or low-density CSLCs.

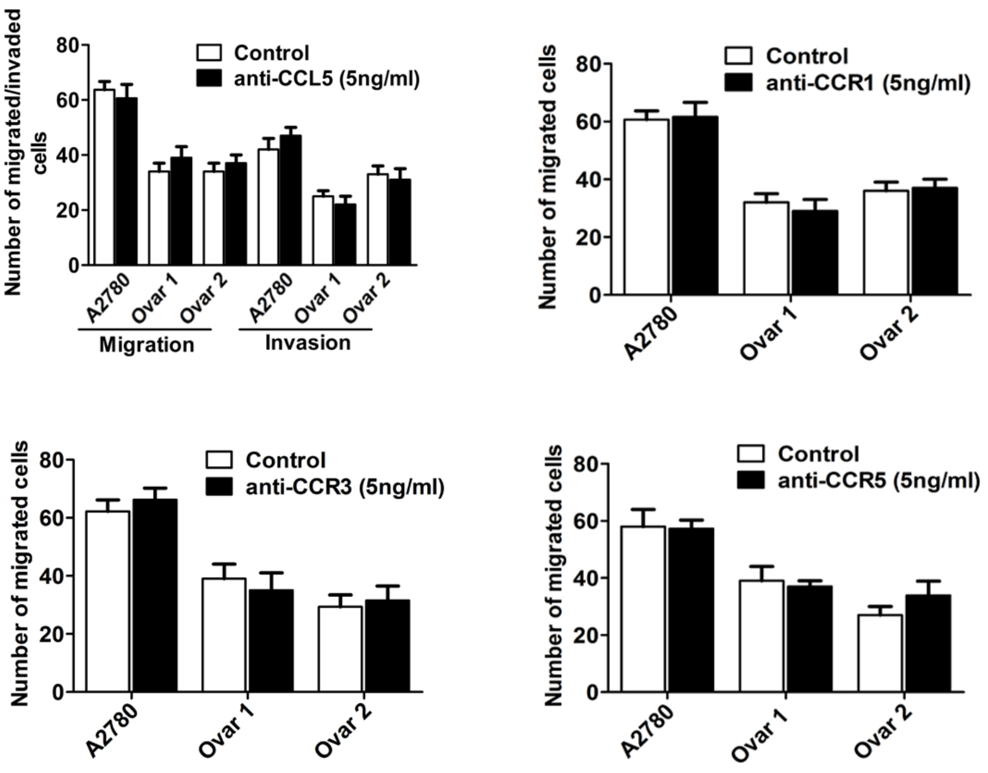

Supplementary Figure 2: The invasion of cancer cells is not influenced by antibodies against CCL5 and its receptors.

NCSLCs derived from A2780, or ovarian cancer patients were treated with antibodies against CCL5 and its receptors for 24 h. The number of migrated cells was determined as described in the Methods section. The error bars represent the means ± standard deviation (SD). Each experiment was repeated at least three times.

Supplementary figure 3

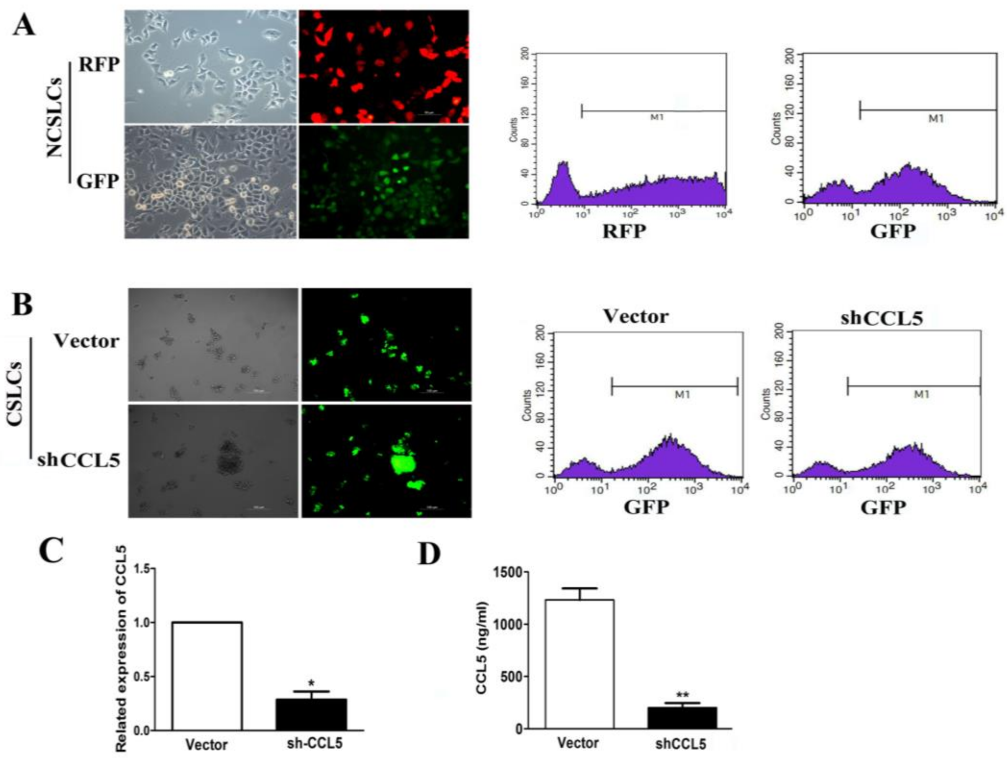

Supplementary Figure 3: Transduction of CSLCs with CCL5-shRNA.

NCSLCs were transduced with RFP or GFP control vector for different purposes (A). CSLCs were transduced with control shRNA or CCL5-shRNA (**B**) and CCL5 gene expression levels determined by real-time PCR (**C**) and ELISA (**D**). The bars correspond to the means ± SD. Each experiment was repeated at least three times. $*P < 0.05$.

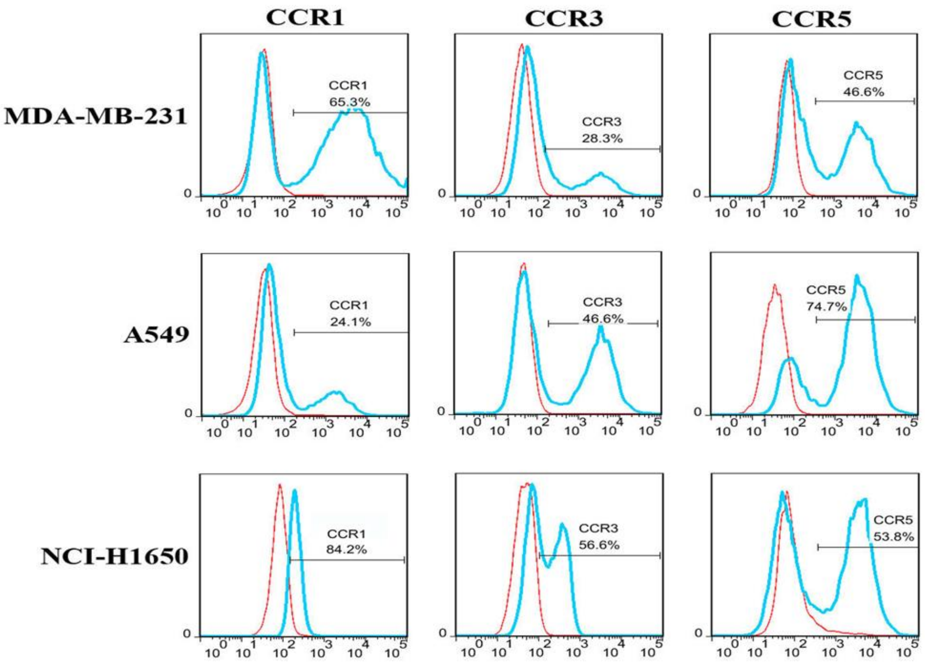

Supplementary Figure 4: Expression of CCR1, CCR3, and CCR5 by MDA-MB-231, A549 and NCI-H1650 cells.

Cell-surface expression of CCR1, CCR3, and CCR5 in breast cancer (MDA-MB-231) and lung cancer (A549 and NCI-H1650) cell lines was assessed by flow cytometry. Each experiment was repeated at least three times.

Supplementary Figure 5

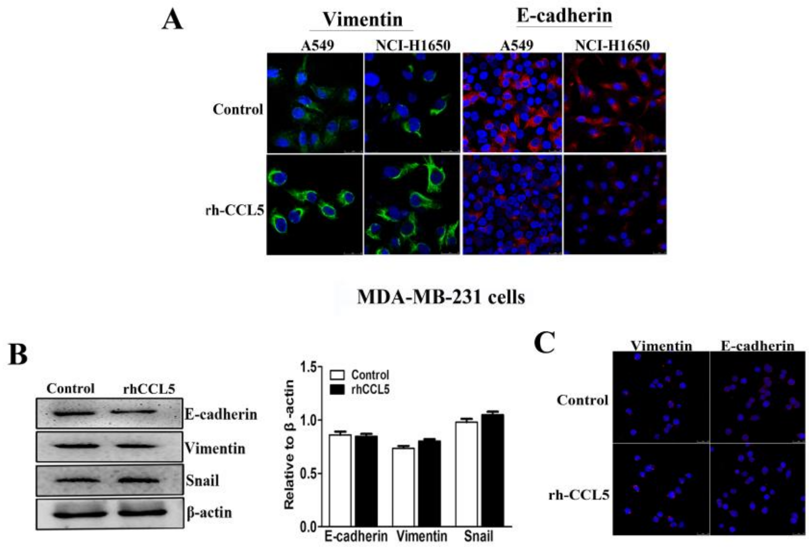

Supplementary Figure 5: CCL5 induces the acquisition of EMT markers by A549 and NCI-H1650 cells, but not MDA-MB-231 cells.

The expression of EMT markers on MDA-MB-231 (**B and C**), A549 (**A**), and NCI-H1650 (**A**) cells in the presence or absence of 5 ng/ml rhCCL5 was assessed. The EMT markers were analyzed by immunofluorescence (**A and C**) and Western blot (**B**). The bars correspond to the means ± SD. Each experiment was repeated at least three times.

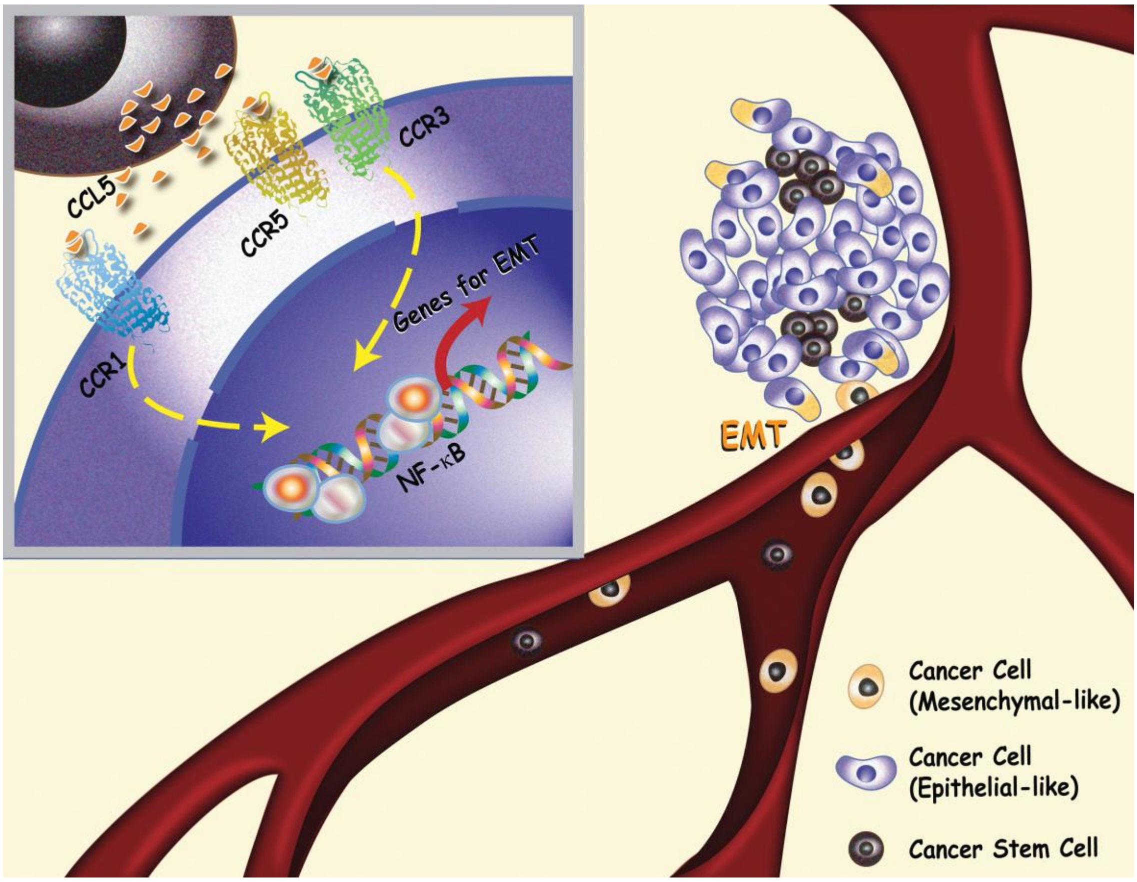

Supplementary Figure 6: Schematic of proposed mechanism for CSLCs promoted migration and invasion of NCSLCs, which depends on the CCL5:CCR1/CCR3/CCR5-NF-κB signaling axis.
